# Picosecond laser to treat complex pigmentary disorders

**DOI:** 10.1016/j.jdcr.2025.02.040

**Published:** 2025-03-21

**Authors:** Hasina Maredia, Saranya P. Wyles, Hafsa M. Cantwell, Julio C. Sartori-Valinotti

**Affiliations:** a Department of Dermatology, Mayo Clinic, Rochester, Minnesota; b Center for Aesthetic Medicine and Surgery, Mayo Clinic, Rochester, Minnesota

**Keywords:** complex medical dermatology, drug-induced hyperpigmentation, hydroxychloroquine-induced hyperpigmentation, hyperpigmentation, lichen planus pigmentosus, picosecond laser

## Introduction

Treatment of pigmentary conditions with picosecond laser has primarily been reported for melasma, solar lentigines, and pigmented congenital lesions such as nevus of Ota.^1^^,^^2^ There is limited literature on the use of picosecond laser among other pigmentary disorders and drug-induced hyperpigmentation, which are often refractory to topical and systemic treatments.^3^^,^^4^ The purpose of this case report is to raise awareness of the positive responses and tolerability of picosecond laser in the management of complex pigmentary disorders, namely lichen planus pigmentosus (LPP) and hydroxychloroquine-induced hyperpigmentation (HH).

## Methods

This retrospective case report evaluated 2 patients seen at Mayo Clinic, Rochester, Minnesota and treated with picosecond laser at the Center for Aesthetic Medicine and Surgery. All patients provided written consent to use their photos.

## Cases

### Case 1

**Fig 1:**
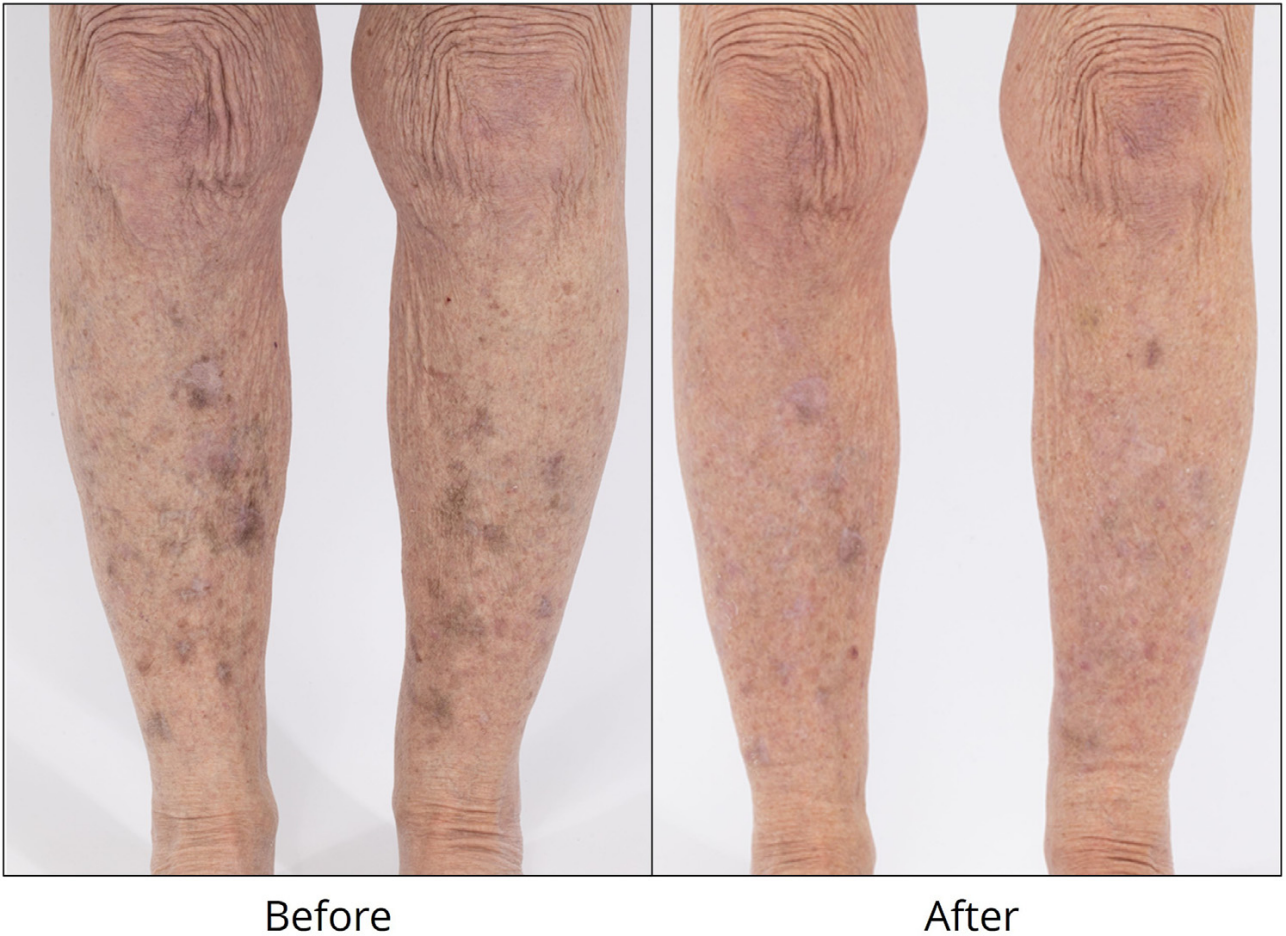
Hydroxychloroquine-induced hyperpigmentation on the anterior aspect of the lower legs was treated with 532 nm, 750 picosecond laser, with results shown after 1 treatment.

A 71-year-old woman, with Fitzpatrick skin type II and history of mixed connective tissue disease, presented with hyperpigmentation over the forearms and shins secondary to chronic hydroxychloroquine. Hydroxychloroquine 200 mg twice daily was started approximately 17 years ago. Over the prior 8 years, she experienced easy bruising followed by erythematous, pruritic patches over the bilateral forearms and anterior lower legs that gradually darkened into red-brown patches. Biopsies showed lichenoid dermatitis, suspected to be secondary to hydroxychloroquine, which was discontinued. Her dyschromia consisting of lichenoid dermatitis with postinflammatory hyperpigmentation was irreversible with hydroxychloroquine cessation. Failed topical treatments included betamethasone dipropionate 0.05% cream, triamcinolone 0.1% cream, and tacrolimus 0.1% ointment. Sun protection and picosecond laser treatment were advised. The affected areas were treated with 532 nm, 750 picosecond laser (Enlighten, Cutera) using 0.5 J/cm^2^ fluence, 8 mm spot size, and 2 Hz to an end point of confluent, mild whitening. She reported 85% resolution of her hyperpigmentation after a single treatment (Fig 1). There has been near-complete resolution after 2 treatments without recurrence while continuing hydroxychloroquine.

### Case 2

**Fig. 2:**
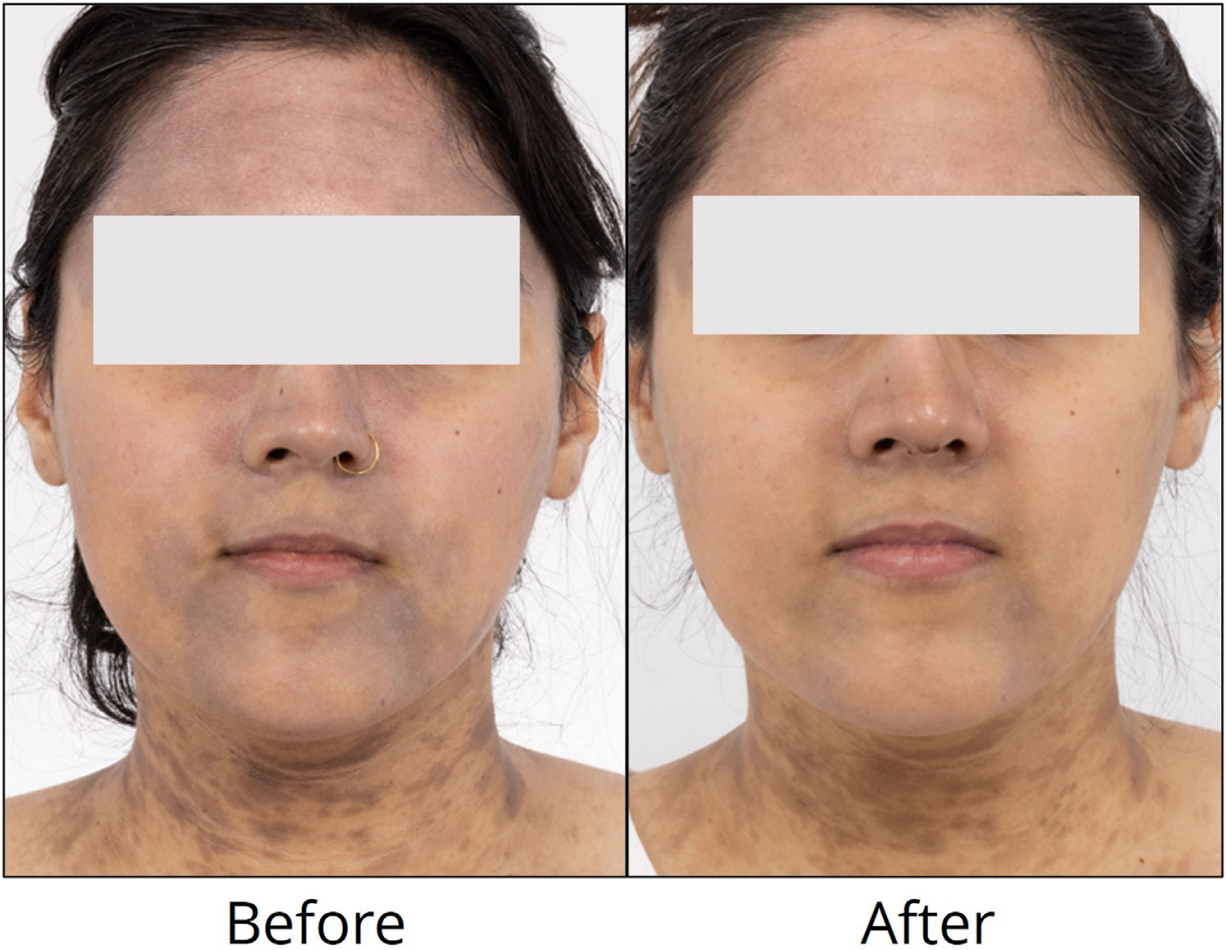
Lichen planus pigmentosus involving the face was treated with a 1064 nm, 750 picosecond laser, with results shown after 5 treatments.

A 31-year-old woman, with Fitzpatrick skin type IV and history of type 1 diabetes, presented with LPP involving the face, neck, and upper extremities. Her lesions were refractory to topical fluocinolone 0.01%-hydroquinone 4%-tretinoin 0.05% cream and 1 month of oral dapsone. Skin biopsy showed pigment incontinence and dermal melanophages without active inflammation, consistent with LPP. Picosecond laser combined with sun protection and topical pimecrolimus were recommended. She deferred systemic treatment options due to plans to conceive in the near future. The affected areas were treated with a 1064 nm, 750 picosecond laser using 0.5 J/cm^2^, 8 mm spot, and 5 Hz with 1 to 2 passes at 4 to 6 week intervals with end point of mild erythema. She completed 6 treatments of the face and neck and 5 treatments of the dorsal hands. Due to concerns of possible koebnerization on the dorsal hands versus worsening hyperpigmentation in the setting of increased sun exposure, laser passes were limited to 1 without subsequent worsening. She reported at least 35% improvement (Fig 2).

There was no new hyper- or hypopigmentation, scarring, or other adverse event in either case.

## Discussion

In this report, picosecond laser was found to be a well-tolerated treatment with positive results for both HH and LPP. For complex pigmentary conditions such as HH and LPP, Q-switched laser has been used in the literature, but there are limited reports on the use of picosecond laser.^1^^,^^2^ Picosecond laser uses photoacoustics to deliver energy with an even shorter pulse duration compared with a Q-switched laser with large spot size and low fluence. Short pulse duration leads to less damage to surrounding tissue and minimal thermal heat, further reducing risk of hyperpigmentation as seen in comparative studies for other pigmentary disorders.^1^^,^^2^ For instance, with nevus of Ota-like macules, picosecond laser had improved clinical outcomes with less discomfort compared with Q-switched Nd:Yag laser.^2^ Picosecond laser also provides a safer option among patients with skin of color.^2^

For the HH case, due to the patient’s lighter skin type, a 532 nm wavelength was selected, versus for LPP in a patient with skin of color, the 1064 nm wavelength was selected. Other considerations in the selection of wavelength are depth of pigment deposition, warranting test spots to determine individualized settings.^2^ Low fluency was used in both cases to decrease risk of hyperpigmentation. Although koebnerization was a possible side effect in the LPP case, there was no worsening with reduction of passes. There were no other adverse effects reported.

Hydroxychloroquine is a first-line treatment option for several connective tissue diseases and other autoinflammatory and autoimmune conditions.^4^ HH, which is an idiosyncratic medication reaction, may lead to discontinuation of hydroxychloroquine despite adequate management of underlying conditions.^4^ Topical treatments are often not feasible nor cost effective as monotherapy for HH. Our case demonstrated the utility of using the 750 nm picosecond laser successfully without recurrence, even with the patient continuing hydroxychloroquine.

LPP is a variant of lichen planus that presents as hyperpigmented patches that occur over sun-exposed areas including the face, arms, and chest.^3^ LPP is difficult to treat, with limited efficacy of topical steroids and oral agents such as dapsone, isotretinoin, and hydroxychloroquine.^3^ LPP has a predilection for female patients in their 30s to 50s, further limiting options for treatment during childbearing years. It is associated with skin of color and diabetes, posing risks of hyperpigmentation and wound healing, respectively, with invasive treatments. It is thus important to have short-term, minimally invasive treatment options for LPP that are safe in darker skin types such as the picosecond laser.

Skin sequelae can have a profound impact on the psychosocial wellness of patients.^5^ Patient-based surveys on radiation tattoos have found that most patients have had negative feelings associated with radiation tattoos, and laser tattoo removal can help improve quality of life.^5^ Recognition of treatment options such as picosecond laser for complex pigmentary conditions can similarly greatly improve self-esteem and quality of life of patients with affected skin refractory to other treatments. Successful treatment with picosecond laser can also enable patients to continue hydroxychloroquine, avoiding other higher risk immunosuppressive medications. Further larger scale, prospective studies on picosecond laser for complex pigmentary conditions, including comparative studies with Q-switched laser, are warranted given its promising potential demonstrated in these cases.

## Conflicts of interest

None disclosed.
